# DCE-MRI for Early Prediction of Response in Hepatocellular Carcinoma after TACE and Sorafenib Therapy: A Pilot Study

**DOI:** 10.5334/jbsr.1278

**Published:** 2018-04-20

**Authors:** Kazuhiro Saito, Joseph Ledsam, Katsutoshi Sugimoto, Steven Sourbron, Yoichi Araki, Koichi Tokuuye

**Affiliations:** 1 Tokyo Medical University, JP; 2 University of Leeds, GB

**Keywords:** DCE-MRI, Tracer kinetic modeling, Sorafenib, TACE, Hepatocellular carcinoma

## Abstract

**Objective:**

Dynamic contrast-enhanced MRI (DCE-MRI) can measure the changes in tumor blood flow, vascular permeability and interstitial and intravascular volume. The objective was to evaluate the efficacy of DCE-MRI in prediction of Barcelona Clinic Liver Cancer (BCLC) staging B or C hepatocellular carcinoma (HCC) response after treatment with transcatheter arterial chemoembolization (TACE) followed by sorafenib therapy.

**Methods:**

Sorafenib was administered four days after TACE of BCLC staging B or C HCC in 11 patients (21 lesions). DCE-MRI was performed with Gd-EOB-DTPA contrast before TACE and three and 10 days after TACE. DCE-MRI acquisitions were taken pre-contrast, hepatic arterial-dominant phase and 60, 120, 180, 240, 330, 420, 510 and 600 seconds post-contrast. Distribution volume of contrast agent (DV) and transfer constant Ktrans were calculated. Patients were grouped by mRECIST after one month or more post-TACE into responders (complete response, partial response) and non-responders (stable disease, progressive disease).

**Results:**

DV was reduced in responders at three and 10 days post-TACE (p = 0.008 and p = 0.008 respectively). DV fell in non-responders at three days (p = 0.025) but was not significantly changed from pre-TACE values after sorafenib. Sensitivity and specificity for DV 10 days post-TACE were 88% and 77% respectively.

**Conclusion:**

DV may be a useful biomarker for early prediction of therapeutic outcome in intermediate HCC.

## Introduction

Sorafenib is a multi-kinase inhibitor drug that provides clinical benefit advanced hepatocellular carcinoma (HCC). It lengthens the duration of stable disease impacting both real and quality-adjusted life years [1]. However, it is an expensive therapy and the prevalence of adverse effects is relatively high. Early prediction of response could help avoid unnecessary patient suffering and offer additional economic benefits.

The combination of sorafenib with transcatheter arterial chemoembolization (TACE) has revealed good therapeutic results [2,3,4,5]. TACE is the standard therapy for intermediate HCC cases where surgery is not possible or is contraindicated. Because TACE enhances the production of angiogenic factors such as vascular endothelial growth factor (VEGF), it increases the possibility of metastasis and recurrence [6]; administering a course of sorafenib alongside TACE can reduce this risk. This presents a problem for follow up: TACE response is usually evaluated on dynamic CT or MRI studies and local recurrence by the presence of enhancement on arterial phase. Standard dynamic studies are unable to evaluate the effect of molecular target therapies such as sorafenib. There is a clinical need for both evaluation and early prediction of HCC response after the combination of sorafenib and TACE.

Functional imaging methods can help solve these problems. Dynamic contrast-enhanced MRI (DCE-MRI) appears to be well correlated with immunohistochemical findings of antiangiogenic, antiproliferative and proapoptotic effects [7]. DCE-MRI can measure the changes in tumor blood flow, vascular permeability and interstitial and intravascular volume that result from these effects.

We evaluate the efficacy of tracer kinetic modelling of DCE-MRI in the early prediction of intermediate HCC response after treatment with TACE followed by sorafenib therapy.

## Materials and Methods

This prospective study was institutional review board approved (Tokyo Medical University, #1149) and informed consent was obtained.

### Subjects

Eleven patients (8 male, 3 female: mean age 65 years) with 21 hepatocellular carcinoma lesions (mean ± standard deviation largest dimension: 22.6 ± 17.9 mm) were included in this study. Tumors were diagnosed pathologically and/or based on the criteria of the American Association for the Study of Liver Disease using abdominal dynamic CT, dynamic MRI using gadolinium-ethoxybenzyl-diethylenetriamine pentaacetic acid (Gd-EOB-DTPA) or contrast-enhanced US [8]. A maximum of two lesions were included per patient to avoid sampling bias. The two largest HCC lesions were chosen if more than two lesions were present. The study inclusion criteria were an initiation on sorafenib therapy, Barcelona Clinic Liver Cancer (BCLC) staging B or C, no contraindications to MRI, and an interval between DCE-MRI before TACE and TACE of no greater than two weeks. Patients were excluded if they were eligible for surgical or locoregional therapy, or if life expectancy was less than 12 weeks.

### Therapy

All patients underwent TACE, followed after four days by sorafenib therapy. The initial dose of sorafenib was 400mg daily and this was increased by oncology physicians if no adverse effects were experienced. TACE were performed using an epirubicin hydrochloride (Nippon kayaku, Tokyo, Japan)-lipiodol (an ethyl ester of iodinated poppyseed oil fatty acids) emulsion or miriplatin hydrate (Miripla®; Dainippon Sumitomo Pharma, Co. Ltd., Osaka, Japan)-lipiodol suspension. Epirubicin-lipiodol emulsion consisted of 18–70 mg epirubicin and 4–10 ml of lipiodol in 10 cases. Miriplatin hydrate-lipiodol suspension was 140 mg miriplatin and 10 ml lipiodol in a remaining case. The dose was determined by the tumor size, number of lesions and liver function [9]. Gelatin sponge particles (Gelpart®; Nippon Kayaku, Tokyo, Japan) followed the injection of the emulsion until stoppage of the feeding arterial flow. Embolic material was injected via segmental artery, right or left hepatic arteries.

### DCE-MRI

**Figure 1:**
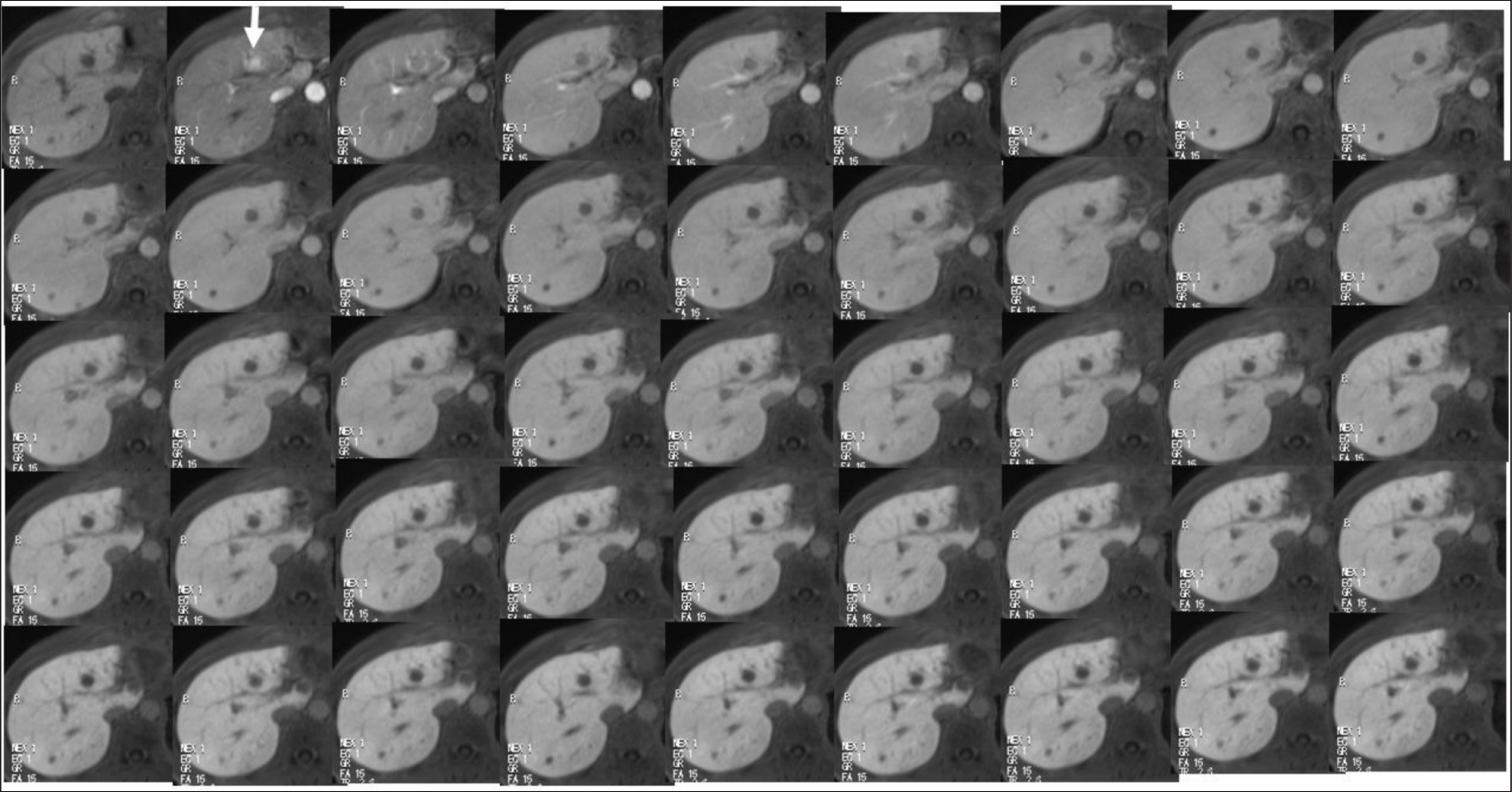
DCE-MRI acquisitions of five images over 30 seconds in each phase were taken pre-contrast, at the hepatic arterial-dominant phase and at 60, 120, 180, 240, 330, 420 510 and 600 seconds post-contrast.

MR imaging was performed with a 1.5 Tesla (T) scanner 32-channel coil system (Avanto, Siemen Medical Systems, Erlangen, Germany) with a peak slew rate of 200 T/m/s. DCE-MRI was performed with T1-weighted 3D gradient echo sequence with fat saturation and volumetric interpolated breath-hold examination (VIBE; Siemens). The sequence parameters were as follows: TR/TE = 2.64/0.98 ms, slice thickness 3 mm, field of view = 400 mm × 68.8%, effective matrix size = 320 × 70%, signal averages = 1, acquisition time = 6 seconds, k-space trajectory, linear filling. DCE-MRI was performed pre-TACE and three and 10 days after TACE. Gadolinium-ethoxybenzyl-diethylenetriamine pentaacetic acid (Gd-EOB-DTPA; Primovist, Bayer) of 0.025 mmol/kg was injected at 2 ml/s via the antecubital vein. DCE-MRI acquisitions of five images over 30 seconds in each phase were taken pre-contrast, at the hepatic arterial-dominant phase and at 60, 120, 180, 240, 330, 420 510 and 600 seconds post-contrast Figure 1.

### Follow-up

Follow-up imaging was performed with dynamic CT, MRI or CT during hepatic arteriography after one month or more post-TACE. The perfusion MRI was used for evaluating outcome at three and 10 days post-TACE. Dynamic CT was performed with either a 16- or 64-detector row CT. Contrast agent (Iohexol 300, Daiichi-Sankyo, Tokyo, Japan) was injected over 30 s [10]. The amount of contrast agent used was 600 mgI/kg [11]. The arterial-dominant phase was obtained using a monitor scan; following this the portal-dominant phase and equilibrium phase were obtained at 60 seconds and four minutes after injection of contrast media. Dynamic MRI was performed using Gd-EOB-DTPA. Gd-DTPA was injected at a rate of 2 mL/s via the antecubital vein, followed by 40 mL of physiological saline. The total amount of contrast media used was 0.025 mmol/kg. Dynamic study included the hepatic arterial-dominant phase, portal-dominant phase, and period four minutes after injection of the contrast material. We used a 3D-VIBE with the dynamic study, with the following parameters: TR/TE, 4.28/1.78 ms; flip angle, 15°; matrix 256 × 217; PAT factor, 2; slice thickness, 3 mm; and acquisition time, 20s. The monitoring scan technique (Care Bolus method) was used to obtain the optimal arterial phase. The hepatobiliary phase was obtained by 3D-VIBE 20 minutes after injecting the contrast material. Angiography-assisted CT was performed with an angiography-combined 16-detector row CT system (Advantx ACT; GE Medical Systems, Milwaukee, WI). CTHA was performed six seconds after the injection of contrast material through a catheter in the common hepatic artery or proper hepatic artery. A total of 10–30 mL of contrast material (Iomeprol 350 mgI/mL; Eisai, Tokyo, Japan) was injected at a rate of 0.8–1.5 mL/s. CTHA was carried out in three phases. Immediately after finishing the first phase, the second phase was obtained, and the third phase was obtained two minutes after beginning the injection of contrast material. The parameters for CT acquisition were as follows: table speed, 13.7 mm/0.5s; collimation, 10 mm; and reconstruction, 5 mm.

Based on this the modified response evaluation criterion in solid tumors (mRECIST) was used to group lesions into responders (complete response (CR) and partial response (PR)) and non-responders (stable disease (SD) and progressive disease (PD)) [12]. Each category was defined as follows:

CR = Disappearance of any intratumoral arterial enhancement in all target lesions.

PR = At least a 30% decrease in the sum of diameters of viable (enhancement in the arterial phase) target lesions, taking as reference the baseline sum of the diameters of target lesions.

SD = Any cases that do not qualify for either PR or PD.

PD = An increase of at least 20% in the sum of the diameters of viable (enhancing) target lesions, taking as reference the smallest sum of the diameters of viable (enhancing) target lesions recorded since treatment started.

### Post-processing

Post-processing was performed by one of the authors with four years’ experience in quantitative DCE-MRI analysis using the software PMI 0.4 [13], blinded to the lesion mRECIST category.

Arterial input functions were defined semi-automatically over five slices inside the abdominal aorta. For each patient a plasma flow map was calculated to aid region of interest (ROI) selection [14,15]. Regions of interest were selected for manually for lesions on the same plasma flow map, taking the whole lesion volume in each region. Each lesion ROI was drawn on all slices where the lesion was visible under the guidance of a senior hepatobiliary radiologists with more than 10 years’ experience.

Concentrations were calculated as the relative signal enhancement S(t)/S0-1 [16], where S(t) is the post-contrast signal intensity and S0 the pre-contrast signal intensity. A fixed haematocrit of 45% was assumed to convert arterial blood concentrations into plasma concentrations. A one-compartment Tofts model was applied to the regions and the perfusion parameters distribution volume of contrast agent (DV) and transfer constant Ktrans were calculated [17,18].

Blood levels of angiogenesis factors angiopoetin (ang2), vascular endothelial growth factor (VEGF) and CKIT were measured in each patient [19] pre-TACE and three and 10 days post-TACE.

### Statistical analysis

Changes in parameter values after sorafenib was compared between the responder and non-responder groups according to the mRECIST criteria. Responder lesions were those classified as CR or PR; non-responder lesions were those classified as SD or PD.

Data was analyzed using the software SPSS and Microsoft Excel. Changes in perfusion parameters DV and Ktrans at pre-TACE, three days after TACE, and 10 days after TACE were analyzed using the Friedman significance test. Differences in perfusion parameters between responder and non-responder groups were evaluated using the Manny-Whitney U test. The Spearman’s Rank Correlation test was performed for all perfusion parameters and angiopoetin2, VEGF and the proto-oncogene CKIT. As many patients had two lesions, the same angiogenesis factor values were assigned to each lesion in a single patient. Analysis of Variance calculations were used to evaluate changes in angiogenesis factors across time. Sensitivity, specificity, negative predictive value (NPV) and positive predictive value (PPV) were calculated for DV as a predictive test. The cut-off for predicting whether a lesion would or would not respond was mean plus two standard deviations of all lesions that responded according to the mRECIST criteria. P values of less than 0.05 were considered significant.

## Results

The range of the intervals between DCE-MRI before TACE and TACE were one to eight days. The interval was 2.7 ± 2.0 days (mean ± standard deviation).

Therapeutic outcome on a per lesion basis was shown in Table 1. The numbers of the lesions classified as CR, PR, SD and PD were 5, 3, 11 and 2, respectively. The eight responder lesions occurred in five patients and the 13 non-responder lesions occurred in eight patients. Two patients had both a responder and non-responder lesion.

**Table 1:** Patients’ demographic data.

Patient	gender	age	segment	Size (mm)	Outcome at 3 days post-TACE	Outcome at 10 days post-TACE	Final outcome	Interval between pre-TACE MRI and TACE (day)	Interval between TACE and follow-up imaging (day)	Type of follow-up imaging

1	f	79	8	56	PR	PR	SD	1	74	CTHA
			8	21	SD	SD	SD			
2	m	75	6	14	CR	CR	CR	8	73	CT
			3	9	SD	CR	CR			
3	m	66	4	10	SD	SD	SD	1	40	CT
			3	20	CR	CR	PR			
4	m	73	7	11	CR	CR	SD	1	114	CT
			3	10	CR	CR	SD			
5	f	69	7	7	CR	CR	SD	1	87	CT
			5	8	CR	CR	SD			
6	m	60	8	80	PR	PR	PD	3	51	MRI
7	f	56	4	15	CR	CR	CR	1	72	CT
			1	16	CR	CR	CR			
8	m	68	7	44	PR	PR	PR	4	68	CT
			3	11	CR	CR	SD			
9	m	71	7	11	CR	CR	PD	4	58	CT
			3	31	CR	CR	SD			
10	m	42	4	18	SD	SD	SD	3	71	CT
			3	17	SD	SD	SD			
11	m	56	6	36	PR	PR	PR	3	71	CTHA
			1	29	CR	CR	CR			

f: female, m: male, CR: complete response, PR: partial response, SD: stable disease, PD: progress disease. TACE: Transcatheter arterial chemoembolization. CTHA: CT during hepatic arteriography. Gray shade indicates the presence of the opposite therapeutic outcome lesion per patient basis.

**Figure 2:**
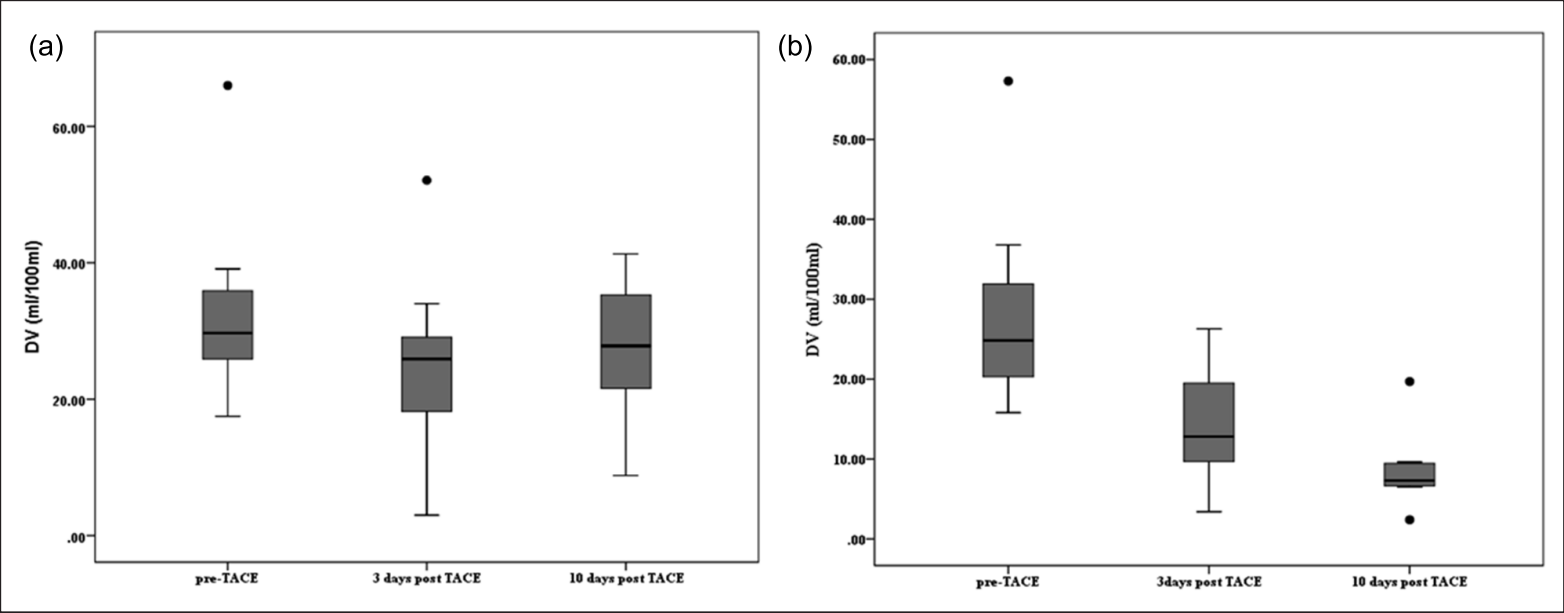
**(a)** Responder lesions, **(b)** non-responder lesions. DV was significantly reduced in all patients after initial TACE (p < 0.001). In responder lesions DV fell progressively at both 3 days and 10 days post-TACE (p < 0.001). DV in non-responders was not significantly different from pre-TACE values at 10 days (p = 0.025). Boxplot shows as follows. Upper, middle and lower line in the box shows upper quartile (75 percentiles), median and lower quartile (25 percentiles), respectively. Upper whisker = upper quartile + 1.5 × IQR (interquartile range). Lower whisker = lower quartile – 1.5 × IQR (interquartile range). IQR = upper quartile (75 percentiles) – lower quartile (25 percentiles).

DV in the responder group was 28.4 ± 12.4, 14.2 ± 7.0 and 8.6 ± 4.7 (ml/100 ml; mean ± standard deviation) pre-treatment, at three days and 10 days post-TACE, respectively. DV in the non-responder group was 32.3 ± 11.6, 24.5 ± 12.1 and 27.0 ± 9.5 (ml/100 ml, mean ± standard deviation) pre-treatment, at three days and 10 days post-TACE, respectively. DV was significantly reduced in all patients after initial TACE (p < 0.001). Changes in DV values are shown in Figure 2. In responder lesions (eight in total) DV fell progressively at both three days and 10 days post-TACE (p < 0.001). DV in non-responders (13 in total) was not significantly different from pre-TACE values at 10 days (p = 0.025). Fall in DV for responders were significant at all time point combinations (pre-treatment versus three days post-TACE, pre-treatment versus 10 days post-TACE, three days and 10 days post-TACE; p = 0.008, 0.008 and 0.047 respectively). A significant fall was observed for DV in non-responders between pre-treatment and three days post-TACE (P = 0.01); changes in DV for the non-responder group between other time intervals were not significant.

**Figure 3:**
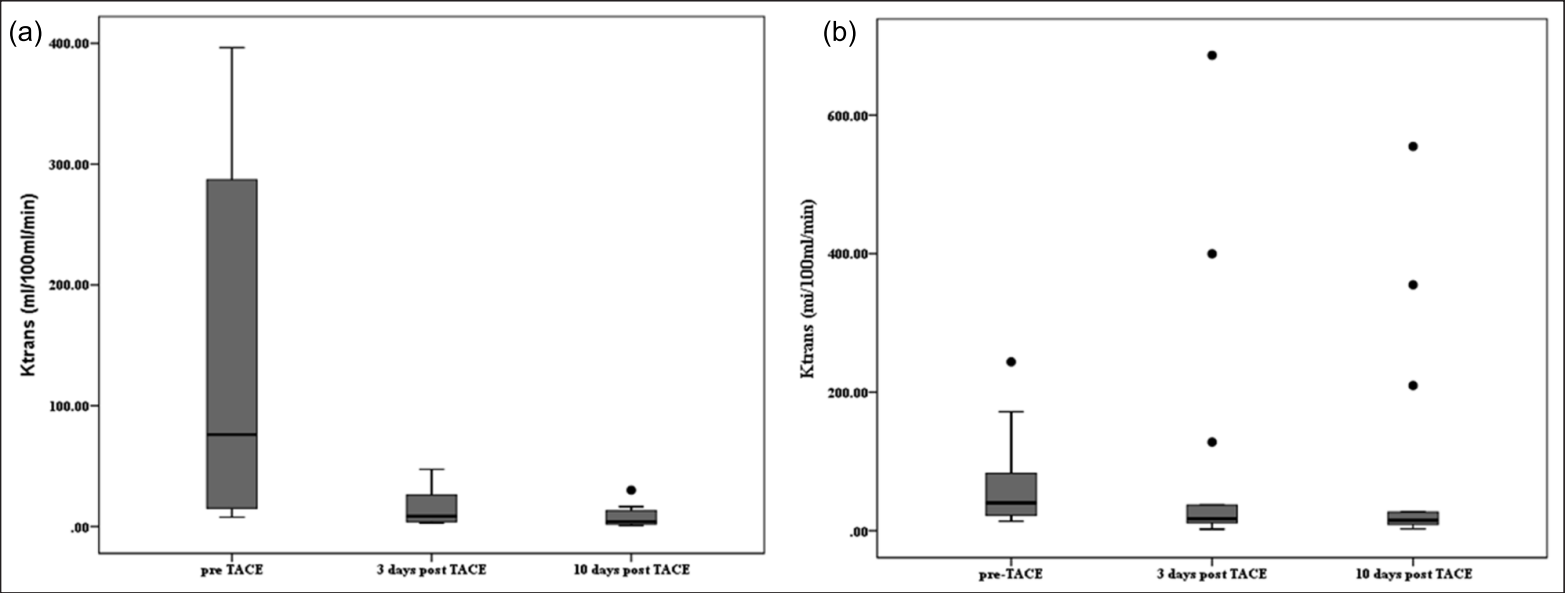
**(a)** Responder lesions, **(b)** non-responder lesions. Ktrans was significantly reduced in the responder group (p = 0.002). Ktrans of responders was significantly reduced between pre-treatment and 3 days post-TACE (p = 0.008) and pre-treatment and 10 days post-TACE (p = 0.016). Responder Ktrans did not significantly change between 3 days and 10 days post-TACE.

Ktrans in the responder group was 145.2 ± 151.3, 15.9 ± 15.3 and 8.7 ± 9.5 (ml/100 ml/min, mean ± standard deviation) pre-treatment, at three days and 10 days post-TACE, respectively. Ktrans in the non-responder group was 67.5 ± 66.5, 105.4 ± 197.3 and 97.0 ± 166.0 (ml/100 ml/min, mean ± standard deviation) pre-treatment, at three days and 10 days post-TACE, respectively. Ktrans was significantly reduced in the responder group (p = 0.002). Changes in Ktrans are shown in Figure 3. The Ktrans of responders was significantly reduced between pre-treatment and three days post-TACE (p = 0.008) and pre-treatment and 10 days post-TACE (p = 0.016). Responder Ktrans did not significantly change between three days and 10 days post-TACE.

There was a significant difference between responder and non-responder groups in DV at three and 10 days post-TACE (p = 0.045 and p < 0.001 respectively), and in Ktrans at 10 days post-TACE (p = 0.029).

Average angiogenesis factor levels at each time point are shown in Table 2. Ang2 decreased from pre-treatment to 10 days by an average of 705 pg/ml in responders and 331 pg/mL in non-responders (p = 0.037). A significant correlation (r = 0.621, p = 0.03) between DV and ang2 was observed. For both VEGF and c-KIT no significant difference was observed between the two groups, and no significant correlations were found with perfusion parameters.

**Table 2:** Average values of angiogenesis factors for responder and non-responder lesions.

Angiogenesis factor	Lesion classification	Pre-TACE	3 days post-TACE	10 days post-TACE	Signifiance of responder vs nonresponder change

Ang2 (pg/ml)	Responder	3663	3913	3208	p = 0.037
	Non-responder	3078	3967	3636	
VEGF (pg/ml)	Responder	69	108	242	p > 0.05
	Non-responder	86	83	201	
c-KIT (ng/ml)	Responder	9.8	7.8	8.2	p > 0.05
	Non-responder	8.5	8.3	8.6	

The sensitivity, specificity and PPV of MR imaging at three and 10 days post-TACE which indicate as responder are 88%, 31% and 44%, and 100%, 31% and 47%. The sensitivity, specificity, NPV and PPV of DV at pre-TACE and three and 10 days post-TACE which indicate as responder are shown in Table 3, along with positive and negative clinical utility indexes, when DV were 40, 30 and 17 (ml/100 ml) or lower [20].

**Table 3:** Sensitivity, specificity, NPV, PPV and clinical utility indexes for DV at pre-TACE and 3 and 10 days post-TACE.

Parameter	Sensitivity	Specificity	PPV	NPV	Positive CUI	Negative CUI

DV pre-TACE	88%	8%	37%	50%	0.322	0.038
DV 3d post-TACE	100%	23%	44%	100%	0.444	0.231
DV 10d post-TACE	88%	77%	70%	91%	0.615	0.699

CUI: clinical utility index.

## Discussion

The perfusion parameter DV fell after TACE therapy in both responder and non-responder groups. Similar changes have been reported by Taouli et al. [21], and may in part be explained by the edematous change of tumor cells and lipiodol accumulation in the sinusoids. This narrows the inter-cellular space and reduces extracellular volume resulting in a fall in overall DV. Another important factor is that TACE reduces arterial flow and prevents contrast media reaching the lesion. In theory flow changes would affect Ktrans but not DV, but as the acquisition time is not sufficiently long to fully characterize the intracellular phase this will artifactually reduce the recorded DV.

DV may be an effective early predictor of response to therapy. DV in the responder group significantly decreased between three and 10 days post-TACE. One reason for this is the added therapeutic effect of sorafenib. Other reports have also shown similar changes in Ve (extravascular extracellular volume) after anti-angiogenic therapy [22,23,24]. Although in theory if a lesion undergoes necrotic change the cellularity would loosen and DV would be expected to increase [22], the anti-angiogenic effect of sorafenib decreases arterial blood flow and DV falls (artifactually, as explained above) as a result. The contrast media was unable to fully perfuse the lesions resulting in apparent decreases in DV. This is not the case in non-responders in whom no significant change in DV was observed at 10 days post-TACE. Reperfusion of the lesions due to insufficient TACE therapy may in part explain this.

The sensitivity and specificity of DV at 10 days post-TACE are shown in Table 4. The cut-off value used was crudely defined mean plus two standard deviations, but resulted in satisfactory predictive performance and clinical utility [20]. Future studies with larger sample sizes are needed to define a suitable cut-off value.

Pre-treatment DV does not appear to be a suitable parameter for predicting response before therapy is commenced. There was no significant difference between pre-treatment DV in responder and non-responder groups. This is consistent with previously reported data using anti-angiogenic drugs for pancreatic cancer [24].

Three pro-angiogenic cytokines were compared with the perfusion parameters but only DV and Ang2 showed significant correlation. One study by Llovet et al. found that VEGF and Ang2 (and c-KIT to a lesser extent) predicted HCC patient survival with sorafenib therapy [19]. Miyahara et al. evaluated the relationship between the eight pro-angiogenic cytokines and the outcome of sorafenib therapy: high expression of Ang2 was associated with poor progression free survival and worse overall survival [25]. The correlation between DV and Ang2 supports this result. Increased vascularity in individual lesions, as expected with higher levels of Ang2, will increase the recorded DV. Such an increase may signal progression of disease and may be useful an early biomarker.

Ktrans values decreased significantly post-therapy in the responder group. Sorafenib reduces capillary permeability through anti-angiogenic effects on tumor microvasculature; this is reflected by the drop in Ktrans (which reflects the capitally permeability-surface area product, blood flow and intracellular uptake rate). Kim et al. reported that Ktrans served as an effective surrogate biomarker for HCC treated with radiation followed by sorafenib therapy [26], and similar results were obtained in this study.

Previous reports on HCC and other primary cancers have found Ktrans to be lower even before treatment in those responding to anti-angiogenic treatment [24,27,28]. Although similar results were obtained in this study, there was no significant difference. This may have been due to the small number of subjects or that patients with stable disease were included in non-responder group. There were several outlier lesions with high Ktrans values (>100 ml/100 ml/min) in the pre-treatment responder group. Insufficient temporal resolution and poor perfusion of some lesions (perhaps due to necrotic components of the lesion) may have led to poor model performance in these cases and affected the parameter estimates.

We hypothesized that Ktrans would correlate with VEGF expression as vascular permeability is regulated by VEGF [29]. VEGF expression is higher in patients with well-differentiated HCC (especially during the transition from portal supply to arterial supply) than in moderately and poorly differentiated HCC [30,31]. In this study VEGF did not correlate with Ktrans, and no significant difference was observed between responder and non-responder groups. All lesions included were hypervascular and more lesions were moderately or poorly differentiated in this work, perhaps explaining why VEGF expression was not so high.

### Limitations

The number of subjects was small. This is the first report of DCE-MRI in evaluation of TACE followed by sorafenib therapy, and we hope future work in this territory will expand our findings to large study populations. The follow-up period was short and overall survival was not evaluated; this study looked only at effect on individual tumor response.

This study has discussed changes in vascularity after TACE and sorafenib therapy. The use of the intracellular contrast agent Primovist means that the parameter DV describes plasma, interstitium and intracellular space. DV will thus be affected by multiple factors such as changes in vasularity, permeability and HCC hepatocyte function. A clearer interpretation of vascularity would require an extracellular contrast agent. This is also the case for interpretation of Ktrans, which may be weighted by both endothelial and intracellular transport rates. However, Ktrans cannot be evaluated with extracellular contrast media. The hepatocyte-specific contrast agent Primovist is now mainly used in evaluating HCC. This contrast agent can detect not only hypervascular HCC but also early HCC, meaning it is well suited for follow-up of HCC during treatment.

The spatial resolution and small size of some tumors made region selection difficult. Effectively treated lesions also show limited contrast enhancement due to poor vascular supply. This has a potential for imperfect identification and selection of lesion dimensions that may bias the results. The temporal resolution was insufficient to fully characterize the contrast agent transport rates. This was a particular problem in measuring Ktrans accurately and the few values >100 ml/100 ml/min recorded provide evidence of this.

In conclusion, DV 10 days post-TACE may be a useful biomarker in early prediction of therapeutic outcome in advanced HCC. Changes in ang2 suggest this may be due to reduced vascular remodeling in non-responding lesions.
